# Li-Doping Effect on Characteristics of ZnO Thin Films Resistive Random Access Memory

**DOI:** 10.3390/mi11100889

**Published:** 2020-09-24

**Authors:** Xiaofeng Zhao, Ping Song, Huiling Gai, Yi Li, Chunpeng Ai, Dianzhong Wen

**Affiliations:** Key Laboratory of Electronics Engineering, College of Heilongjiang Province, Heilongjiang University, Harbin 150080, China; 2181213@s.hlju.edu.cn (P.S.); 2191273@s.hlju.edu.cn (H.G.); 2171256@s.hlju.edu.cn (Y.L.); aichunpeng@hlju.edu.cn (C.A.); wendianzhong@hlju.edu.cn (D.W.)

**Keywords:** resistive random access memory, Li-doping ZnO thin films, resistive switching characteristics, magnetron sputtering

## Abstract

In this study, a Pt/Ag/LZO/Pt resistive random access memory (RRAM), doped by different Li-doping concentrations was designed and fabricated by using a magnetron sputtering method. To determine how the Li-doping concentration affects the crystal lattice structure in the composite ZnO thin films, X-ray diffraction (XRD) and X-ray photoelectron spectroscopy (XPS) tests were carried out. The resistive switching behaviors of the resulting Pt/Ag/LZO/Pt devices, with different Li-doping contents, were studied under direct current (DC) and pulse voltages. The experimental results showed that compared with the devices doped with Li-8% and -10%, the ZnO based RRAM device doped by 5% Li-doping presented stable bipolar resistive switching behaviors with DC voltage, including a low switching voltage (<1.0 V), a high endurance (>10^3^ cycles), long retention time (>10^4^ s), and a large resistive switching window. In addition, quick switching between a high-resistance state (HRS) and a low-resistance state (LRS) was achieved at a pulse voltage. To investigate the resistive switching mechanism of the device, a conduction model was installed based on Ag conducting filament transmission. The study of the resulting Pt/Ag/LZO/Pt devices makes it possible to further improve the performance of RRAM devices.

## 1. Introduction

Recently, resistive random access memory (RRAM) has been considered as a promising candidate for next generation nonvolatile memory (NVM) devices, due to its significant advantages concerning simplicity of structure, low power consumption, a high level of integration, fast read and write, high tolerance and compatibility, etc. [1,2,3,4]. So far, RRAM has been reported in a variety of material systems, including organic materials [5], complex composition materials [6], and binary transition metal oxide materials [7,8]. In addition, compared with the other material systems, various new types of metal-oxide based RRAM devices are being devised, attributable to their potential advantages [9,10], as well as excellent compatibility with the current complementary metal oxide semiconductor (CMOS) technology [11]. Nevertheless, among these metal oxide materials, ZnO which has become the third generation oxide semiconductor material attracts many people to study it, in the field of RRAM, due to exhibiting unique features, i.e., easy preparation processes, a large memory window, and the possibility of fabricating transparent and flexible electronic devices [12].

However, with the diversification of high performance ZnO-based RRAM devices, especially as more and more memory devices require higher storage density, faster read–write speed, a smaller storage unit, and higher integrated compatibility, it is in no doubt that the ZnO-based RRAM devices with low switching voltage, high *I*_ON_/*I*_OFF_ ratio, and uniformity of switching parameters are being pursued [13,14]. To solve the above ZnO-based resistive switching devices problems, many researchers attempted to improve the resistive switching characteristics by doping methods. To optimize the switching performance of ZnO-based RRAM, a lot of research has been carried out in previous studies to investigate the resistive switching characteristics of ZnO thin films doped by various elements [15,16]. For example, Li et al. studied the stability of Ti-doped ZnO devices and analyzed their switching mechanism based on first principles [17]. Meanwhile, Simanjuntak et al. proposed ITO/Zn_1−x_Co_x_O/ITO transparent resistive switching memory devices, achieving a sufficient memory window at an appropriate amount of Co dopant in an ZnO resistive switching layer [18]. In addition, He et al. reported the effects of Pr-doping amount on the resistive switching behaviors of Pt/Zn_1−x_Pr_x_O/Pt memory cells, improving the performance of devices by lowering the c-axis orientation of Pr-doped ZnO thin films, i.e., achieving a good endurance, a long retention, and a uniform switching voltage [19]. Recently, Lin et al. proposed an unipolar Pt/Li_x_Zn_1−x_O/Pt RRAM device controlled by various defect types, indicating that the Li-doping concentration influences the distribution of defect types in the Li_x_Zn_1−x_O films, which is a crucial factor for adjusting the resistance ratio of the device [20]. Based on the above analysis, it can be found that several deficiencies still exist in these works, such as a small resistive switching window, large and dispersed set/reset voltages, and an absence of pulse response, and so on.

In this work, a high performance Pt/Ag/LZO/Pt RRAM with different Li-doping was fabricated using a magnetron sputtering method, and the effects of various Li-doping concentrations on the resistive switching (RS) behaviors of the RRAM devices was also investigated. Additionally, the resistive switching behaviors at DC voltage were researched, including switching voltage, endurance, retention time, and resistive switching window, and so on. To analyze the resistive switching mechanism of the resulting devices, a conduction model was built based on Ag conducting filament transmission. This study on the Pt/Ag/LZO/Pt device makes it possible to fabricate a high performance ZnO-based RRAM device suitable for practical application in the future.

## 2. Materials and Methods

### 2.1. Fabrication of Device

**Figure 1:**
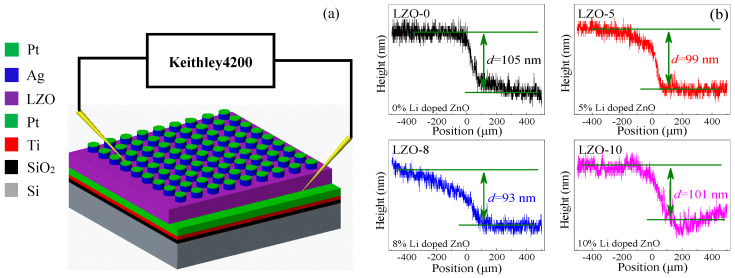
(**a**) The schematics of the Pt/Ag/LZO/Pt RRAM device. (**b**) The thicknesses of the ZnO thin films with different Li-doping contents.

The Pt/Ag/LZO/Pt resistive switching devices were fabricated on SiO_2_/Si substrates using radio frequency (RF) magnetron sputtering (JGP-DZS, Shenyang Sky Technology Development Co., Ltd., Shenyang, China). The main fabrication process and electrical characteristics test system of the proposed devices are as follows in Figure 1. (a) Depositing a Ti film (20 nm) as an adhesion layer on a SiO_2_/Si substrate utilizing a direct current magnetron sputtering method (using a pure Ti target size: D60 mm × 5 mm, purity: 99.99%, ZhongNuo Advanced Material Technology Co., Ltd., Beijing, China), and then depositing a Pt layer (100 nm) on the above Ti/SiO_2_/Si substrate as a bottom electrode (BE) by repeating the above sputtering method (using a pure Pt target size: D60 mm × 5 mm, purity: 99.99%, ZhongNuo Advanced Material Technology Co., Ltd., Beijing, China). (b) Sputtering two resistive switching layers, i.e., a pure ZnO thin film by using a pure ZnO (size: D60 mm × 4 mm, purity: 99.99%, ZhongNuo Advanced Material Technology Co., Ltd., Beijing, China) ceramic target at 200 °C and ZnO thin films with different Li-doping by using a ZnO:Li_2_CO_3_ (ZnO/Li_2_CO_3_, 95/5 wt.%, 92/8 wt.%, 90/10 wt.%, ZhongNuo Advanced Material Technology Co. Ltd., Beijing, China) ceramic target based on the same above sputtering method. (c) Depositing an Ag thin film (100 nm) as the top electrode (TE), with a diameter of 800 μm, to the resulting ZnO thin film surfaces with different Li-doping contents by a metal mask method, and then depositing a Pt layer (100 nm) to the above Ag TE to protect the TE from oxidation, carrying out the sputtering for Ag and Pt in the atmospheric conditions of Ar gas (47 sccm) with a RF power of 100 W and a pressure of 1.0 Pa, however in the mixed atmosphere of argon (47 sccm) and oxygen (15 sccm) at 200 °C with RF power of 220 W and the same pressure for the pure ZnO and Li-doping ZnO. Finally, four kinds of Pt/Ag/LZO/Pt/Ti RRAM device, with different Li-doping ZnO film thicknesses, were prepared under the same preparation process, except for using different Li-doping contents. The fabricated resistive switching devices with Li-doping contents of 0%, 5%, 8%, and 10% were named as LZO-0, LZO-5, LZO-8, and LZO-10, respectively.

To investigate the electrical characteristics of the resistive switching devices, Keithley 4200-SCS semiconductor parameter test systems (Tektronix, Solon, OH, USA) were utilized, exerting a forward biased voltage to the Pt/Ag top electrode and grounding the Pt/Ti bottom electrode. In order to give the thicknesses of the resistive switching layers, a step profiler (NanoMap 500LS, AEP Technology, Santa Clara, CA, USA) was adopted to characterize the test results as shown in Figure 1b. Through calibrating the step profiler with a sample of standard thickness, the thickness of the resistive switching layer could be measured according to the drop of step using the step profiler, taking the average of ten measurements as the thickness of the device. The resulting resistive switching layer thickness of the LZO-0 was 105 nm, those of the LZO-5, LZO-8, and LZO-10 RRAMs were 99 nm, 93 nm, and 101 nm, respectively.

### 2.2. Material Characterizations

**Figure 2:**
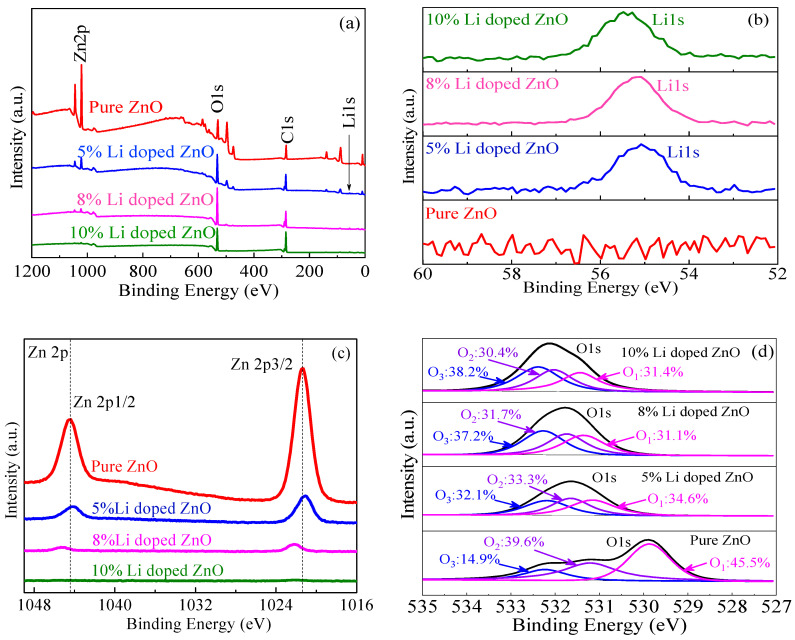
(**a**) X-ray photoelectron spectroscopy (XPS) full survey spectra of pure ZnO, and ZnO thin films with different Li-doping contents; (**b**) Li 1s; (**c**) Zn 2p; (**d**) fitting of O 1s.

To investigate the influences of Li-doping on the chemical composition and valance state of the resulting pure ZnO as well as Li-doping ZnO thin films, X-ray photoelectron spectroscopy (XPS, VG ESCALAB MK II, VG Instruments, Manchester, UK) testing was carried out. A typical wide scan spectrum of the ZnO films is shown in Figure 2a, where all of the elements in the thin films, including Zn 2p, O 1s, Li 1s, and C 1s, are identified by a survey scan in the energy range from 0 to 1200 eV. As seen in Figure 2a, the C 1s peak comes from the adsorbed C on the surface of the sample. Additionally, using software (ThermoFisher SCIENTIFIC, Waltham, MA, USA) to fit the O 1s made it possible to estimate the different types of oxygen percentages for the resulting films by calculating each peak area. Through quantitatively analyzing the Li-doping ZnO thin film surface by the XPS in Figure 2b, the Li concentration in the ZnO thin films with the Li-doping of 0% and 5%, 8%, and 10% can be obtained, i.e., approximate 0 at.%, 21 at.%, 23 at.%, and 26 at.%, respectively. As shown in Figure 2c, the binding energy of the Zn 2p 3/2 and Zn 2p 1/2 in the ZnO thin films doped by different Li-doping contents respectively locates at about 1021.37 eV and 1044.48 eV, with the spin orbit splitting at 23.11 eV, which is consistent with the reported comparison [21]. With the increase of the Li doping concentration, the area of Zn peak will gradually decrease, resulting in a not obvious Zn peak for the 10% Li-doped ZnO films. This is because the Zn vacancies dominate in the defects of low Li-doped ZnO. However, the reduced Zn vacancies due to Li-doping influence the binding energy of Zn 2p, i.e., reducing the binding energy of Zn 2p of 10% Li-doped ZnO. In addition, all of the symmetrical Zn 2p peaks mean no existence of multiple forms of Zn in the sample [22]. Figure 2d shows the fitted O 1s peaks of the pure ZnO and the Li-doped ZnO thin films, which can be fitted into three Gaussian peaks with different binding energies, centered at 530 eV, 531 eV, and 532 eV [20,23], i.e., corresponding to the lowest O_1_, the medium O_2_, and the highest O_3_ peaks, respectively. The medium binding energy O_2_ (at 531 eV), influenced by oxygen-deficient regions within the ZnO matrix, is relative to the concentration of oxygen vacancies, V_O_, in the total oxygen compositions, with V_O_ densities of 39.6% (in the pure ZnO), 33.3% (in the 5% Li-doping ZnO), 31.7% (in the 8% Li-doping ZnO), and 30.4% (in the 10% Li-doping ZnO), indicating the decrease of oxygen vacancies with increasing Li-doping contents. Moreover, due to the existence of a large amount of chemical absorption oxygen on the surface of the thin films, O_3_ can be ignored here.

**Figure 3:**
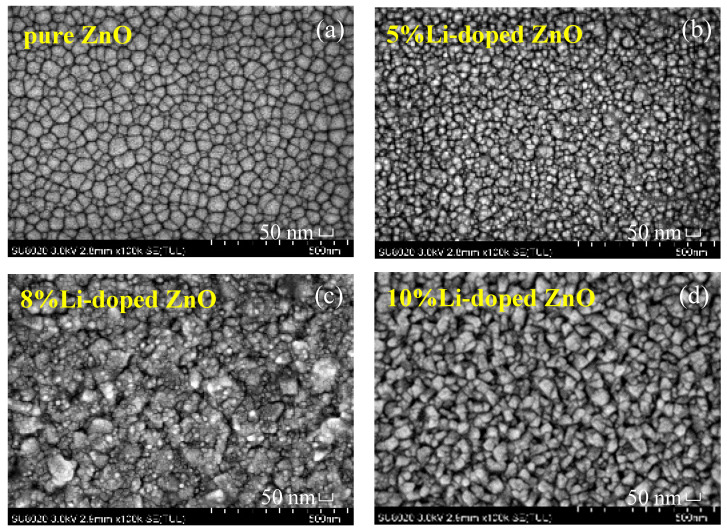
The SEMs of pure and Li-doping ZnO films on the Pt/Ti/SiO_2_/Si substrates: (**a**) pure; (**b**) 5%; (**c**) 8%; (**d**) 10%.

**Figure 4:**
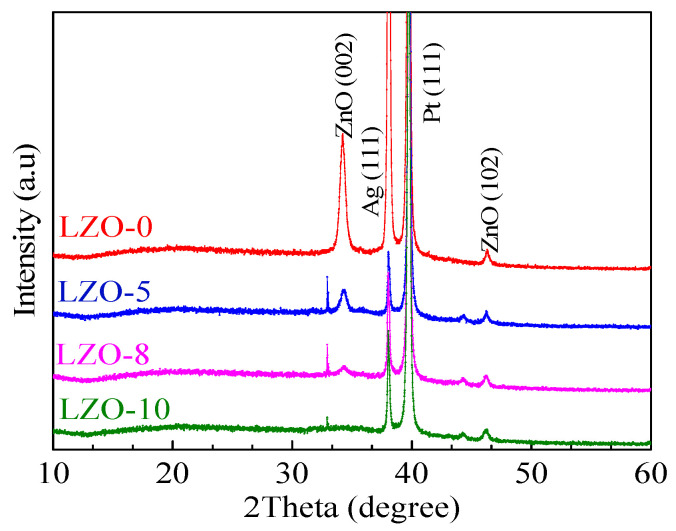
The X-ray diffraction (XRD) spectrum of LZO-0, and LZO-5, LZO-8, and LZO-10 devices.

To observe the surface morphologies for the resulting films on the Pt/Ti/SiO_2_/Si substrates, a field emission scanning electron microscope (FE-SEM, SU8020) test was carried out. From Figure 3a, it can be seen that the smooth surface of the pure ZnO thin film has no cracks, proving the existence of the high packing density in the film. Nevertheless, doping Li into the ZnO thin films resulted in the decrease of the average grain sizes and a much denser microstructure in the films with the increase of the Li-doping contents, as shown in Figure 3b–d. It is common knowledge that a dense microstructure is closely related to a reliable electrical property and uniformity of device. To investigate the effects of Li-doping on the microstructures of the devices, the phase properties of the fabricated devices were characterized by X-ray diffractometer (XRD, Bruker AXS D8 ADVANCE, Billerica, MA, USA) with Cu Kα1 radiation, as shown in Figure 4. The test results give the typical ZnO characteristic peaks corresponding to the (100) and (102) planes (JCPDS No. 36-1451), revealing the existence of a strong (002) orientation wurtzite structure in the devices, and the formation of films with a high orientation [12].

## 3. Results and Discussion

### 3.1. The I–V Characteristics of Devices under DC Voltage

**Figure 5:**
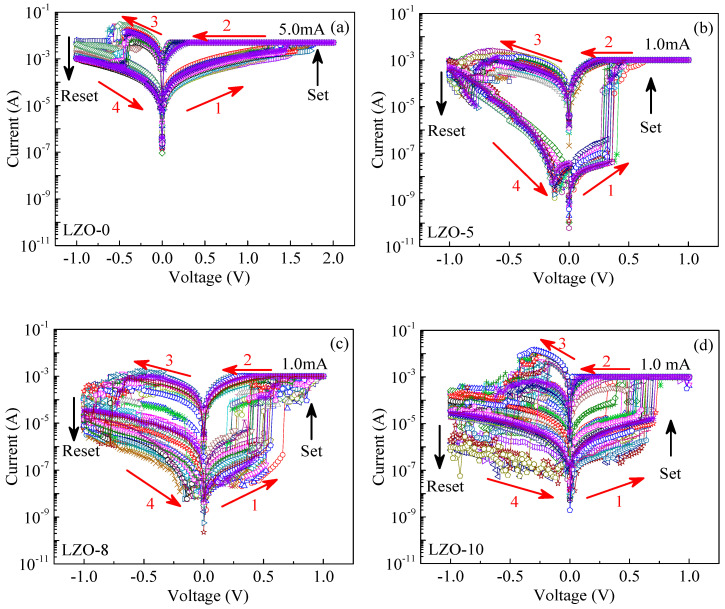
The *I*–*V* curves of resistive switching devices under DC voltages: (**a**) LZO-0; (**b**) LZO-5; (**c**) LZO-8; (**d**) LZO-10.

At room temperature, the resistive switching characteristics of the fabricated devices were measured by a semiconductor parameter analyzer under a direct current (DC) voltage (with the sweeping speed of 25 mV/s). Figure 5a–d show the current–voltage (*I*–*V*) characteristic curves of the LZO-0, LZO-5, LZO-8, and LZO-10 devices with 30 switching cycles, respectively. As shown in Figure 5a, when exerting a biased voltage to the pure LZO-0 device in a sequence of 0 V→2.0 V→0 V→−2.0 V→0 V, the resistive switching state can transform from a high resistance state (HRS) to a low resistance state (LRS) at a high average set voltage of *V*_LZO-0__set_ ≈ 1.5 V, with a very small *I*_ON_/*I*_OFF_, less than 10. When increasing the applied voltage up to a maximum of 2.0 V, and then gradually returning to 0 V, the device remains at a LRS. When applying a negative biased voltage from 0 V to −2.0 V (without a compliance current), the resistance of the device transforms from a LRS to a HRS, becoming a reset operation process. The repeated *I*–*V* results indicated that the LZO-0 device realizes a bipolar resistive switching behavior, with a good resistance uniformity, to transform from a HRS to a LRS at a high average set voltage.

In contrast, a biased sweeping voltage was applied to the Li-doping ZnO devices in a sequence of 0 V→1.0 V→0 V→−1.0 V→0 V, as shown in Figure 5b–d. Initially, all of the Li-doping ZnO devices remained at a HRS under no biased voltage. When exerting a positively biased voltage from 0 V to 1.0 V, a compliance current of 1.0 mA was restricted to prevent the devices from an irreversible breakdown. With respect to the pure device, the LZO-5 device displayed a better bipolar resistive switching performance, with an excellent resistance uniformity, to transform from a HRS into a LRS at a low average set voltage of *V*_LZO-5__set_ ≈ 0.4 V and a high *I*_ON_/*I*_OFF_ more than 10^4^, as shown in Figure 5b, benefiting the application of resistive switching devices in non-volatile memories and logic operations. In addition, when applying a negative voltage from 0 V to −1.0 V (without a limited current), the resistance of the device transformed from a LRS to a HRS at a reset voltage about −0.75 V. As shown in Figure 5c,d, the devices of LZO-8 and LZO-10 achieved obvious bipolar resistive switching behaviors to transform from a HRS into a LRS at corresponding average threshold voltages of *V*_LZO-8set_ ≈ 0.5 V and *V*_LZO-10set_ ≈ 0.6 V, both with *I*_ON_/*I*_OFF_ > 10^2^, significantly less than that of the LZO-5 device. When increasing the applied voltage up to a maximum of 1.0 V, and then gradually returning to 0 V, the devices remained at the LRS. When applying a negative biased voltage from 0 V to −1.0 V, the resistances of the devices transformed from a LRS to a HRS. Based on the above analysis, it can be seen that the set voltages were increased and the resistance uniformity to transform from a HRS into a LRS of the LZO devices was reduced with increasing the doping concentration of Li. Thus, it is possible to compare the LZO-5 device the other devices to present a super resistive switching behavior with the lowest average set voltage and the highest *I*_ON_/*I*_OFF_.

**Figure 6:**
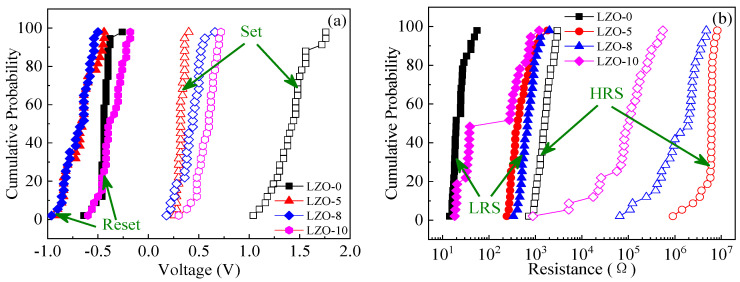
The statistical distributions of resistive switching parameters for the RRAM devices: (**a**) set and reset voltages; (**b**) high-resistance state (HRS) and low-resistance state (LRS) resistances.

Figure 6 shows the statistical distributions of the resistive switching parameters for the devices. Under different Li-doping concentrations, the cumulative possibilities of *V*_reset_ and *V*_set_ for the pure ZnO and LZO devices under 30 switching cycles were plotted, as shown in Figure 6a. As seen from that, the LZO-5 RS device exhibited a low oscillation at *V*_set_ and a narrow distribution between *V*_reset_ and *V*_set_ compared with the other devices, helping to stabilize the operating voltages and reduce the possibility of false operation during the switching. Figure 6b shows the cumulative probability plots of the LRS and HRS resistances for the devices, where the Li-doped ZnO thin film represents a high HRS that significantly increases with Li-doping concentration, resulting in a higher switching current ratio *I*_ON_/*I*_OFF_. Nevertheless, with increase of the Li-doping concentration, the distribution between the HRS and LRS reduces. This indicates that the LZO-5 device displays a better bipolar resistive switching performance compared with the others, i.e., an excellent stability at a low *V*_LZO-5set_ ≈ 0.4 V and a high *I*_ON_/*I*_OFF_ more than 10^4^.

### 3.2. The RS Characteristics of LZO-5 Device under Pulse Voltage

**Figure 7:**
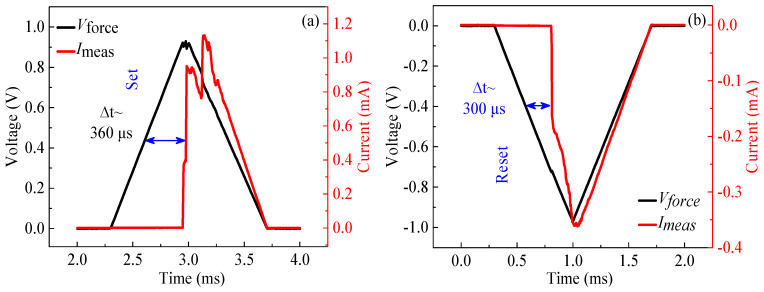
The switching time of the LZO-5 device at a pulse voltage: (**a**) set; (**b**) reset.

According to the previous *I*–*V* characteristics of the LZO devices, the LZO-5 device represented an excellent performance in a DC sweeping mode compared with the others. To further study the resistive switching characteristics of the LZO-5 device, a pulse mode was utilized to explore the switching speed between the HRS and LRS at a set/reset voltage of 1.0 V/−1.0 V with an effective set/reset pulse width of 5 × 10^−6^ s [24,25], as shown in Figure 7. As seen in the set/reset process with an input bias (see black line) and a response current (see red line) in Figure 7a,b, the typical write/erase speed was exhibited under sequence set/reset bias pulses. In addition, the set/reset response time, ∆t, indicates the delay between the response current and input voltage corresponding to the middle of the leading edge, with a set response of ~360 μs under 1.0 V/5 × 10^−6^ s pulse and a reset response of 300 μs under −1.0 V/5 × 10^−6^ s pulse, respectively.

**Figure 8:**
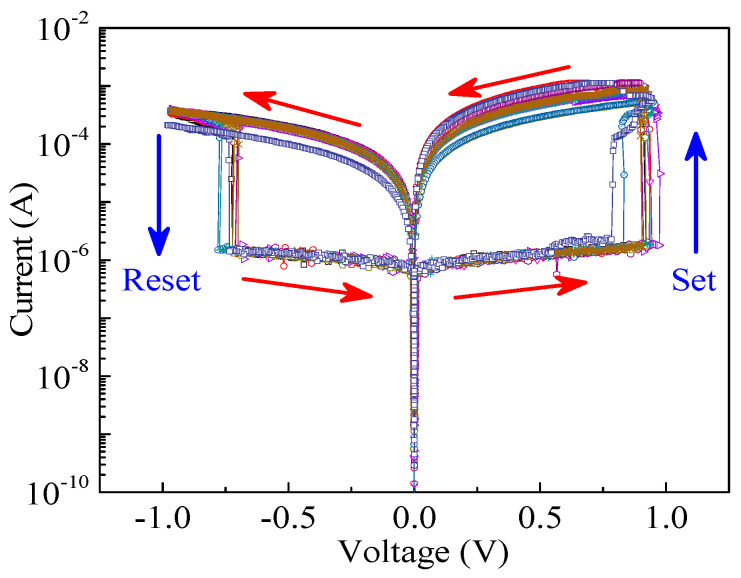
The repeated *I*–*V* characteristic curves under pulse voltage.

By applying a single pulse voltage to the LZO-5 device, the test results show that it is possible to quickly switch between the HRS and LRS, and realize a fast pulse response, contributing to the fast erasing and writing of data. Figure 8 gives the repeated test results with 10 measurement cycles at the same pulse voltage. It can be seen that the device can quickly change from HRS to LRS under the set pulse voltage and from LRS to HRS under the reset pulse voltage, realizing a stable and repeated resistance switching cycle.

**Figure 9:**
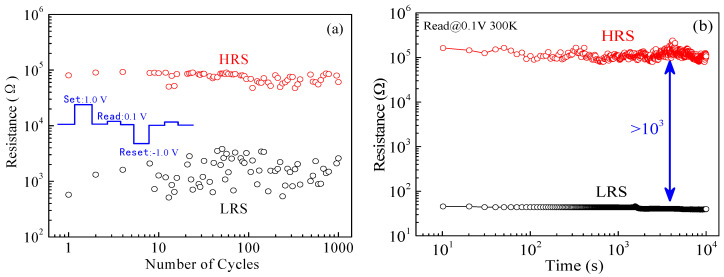
The resistive switching characteristics of the LZO-5 device: (**a**) endurance; (**b**) retention.

It is common knowledge that excellent endurance and long retention are fundamental properties for nonvolatile memory devices, i.e., performing multiple erases/writes of data and retaining the data of the memory device for a long time. Thus, an endurance test was repeated more than 10^3^ cycles, as shown in Figure 9a, in the different operation conditions, including pulse operation with a set voltage of 1.0 V, a reset voltage of −1.0 V, a read voltage of 0.1 V, and a pulse with a width of 5 × 10^−6^ s. It can be seen that the LZO-5 device successfully sustained the write/erase cycles for more than 10^3^ and had no obvious variation during the pulse test, indicating the existence of excellent reliability. Furthermore, a super retention for HRS and LRS can be retained up to 10^4^ s without degeneration as shown in Figure 9b. The above test results and analysis indicate that the LZO-5 device has a good endurance and long retention, much suited to practical applications in nonvolatile memory in the future.

### 3.3. The Switching Mechanism of LZO-0 and -5 Devices

**Figure 10:**
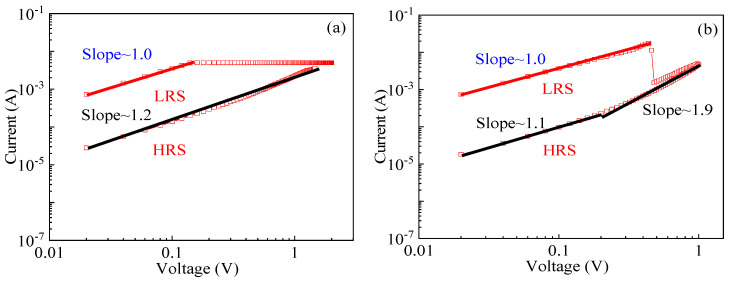

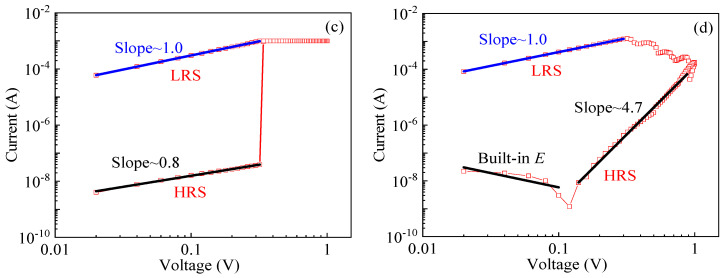
The linear fitting of the *I–V* curve of the LZO-0 and -5 devices in the double logarithmic scale: (**a**) in the positive bias region of LZO-0; (**b**) in the negative bias region LZO-0; (**c**) in the positive bias region of LZO-5; (**d**) in the negative bias region LZO-5.

To understand the conduction mechanisms of the resistive switching devices, the *I–V* characteristic curves of the LZO-0 and LZO-5 devices, under a forward and a reverse bias voltage, were plotted in double-logarithmic scales, as shown in Figure 10a,b. The analog resistive switching mechanisms of the LZO-0 device can be analyzed in Figure 10a. Under a low positive bias, lower than the transition voltage 1.6 V at an HRS, the transport follows Ohm’s law (with slope of ~1.2). When the applied voltage bias exceeds 1.6 V, meaning that the conduction enters a trap-filled-limited region, the traps are gradual filled up and the conduction becomes space-charge-limited, following Child’s law (*I*_Child_ ∝ *V*^2^). Constructively, the *I*–*V* characteristic curve of LRS under the low negative bias, as shown in Figure 10b, is confirmed as an ohmic conduction mechanism after fitting; in addition, its HRS fit straight line has two slopes of 1.1 and 1.9, confirming the ohmic conduction mechanism and space-charge-limited conduction [16], respectively. At this time, the trap in the RS layer has already been filled with injected carriers, and the injected carriers make a major contribution to conduction. Based on the above analysis, due to pure ZnO thin films containing a large amount of oxygen vacancies, the device represents an analog resistive switching behavior attributable to the charge trapping and de-trapping by oxygen vacancies [26].

With respect to that, the fitted two straight lines for the LZO-5 device are shown in Figure 10c, with the slopes of 0.8 and 1.0 at HRS and LRS, respectively. It indicates that the fitting result at HRS conforms with the Schottky emission mechanism (Ln *I* ∝ *V*^1/2^) [27,28], where the direction of the applied electric field is consistent with the direction of the built-in electric field. When increasing the voltage up to 0.4 V, it was found that a nearly vertical current jump can be observed, proving the formation of conductive filaments in the device. Based on the fitting at LRS, the conduction is mainly caused by the thermally excited free electrons, conforming to the ohmic conduction mechanism (*I* ∝ *V*), attributable to that the injected electrons captured by the owning defects of the ZnO layer. 

In Figure 10d, the *I*–*V* characteristic curve of LRS is confirmed as an ohmic conduction mechanism after fitting; HRS’s fits two straight lines, one with a slope of 4.7 and one with a negative slope. Among them, the straight line with a slope of 4.7 is confirmed to be space-charge-limited conduction by fitting [29]. At this time, the trap in the RS layer has already been filled with injected carriers, and the injected carriers make a major contribution to conduction. The straight line with a negative slope is considered to be the presence of a reverse built-in electric field (caused by the different Schottky barrier heights of the top/bottom electrode and the RS layer) [30]. The reverse built-in electric field gradually dominates as the applied negative voltage decreases.

**Figure 11:**
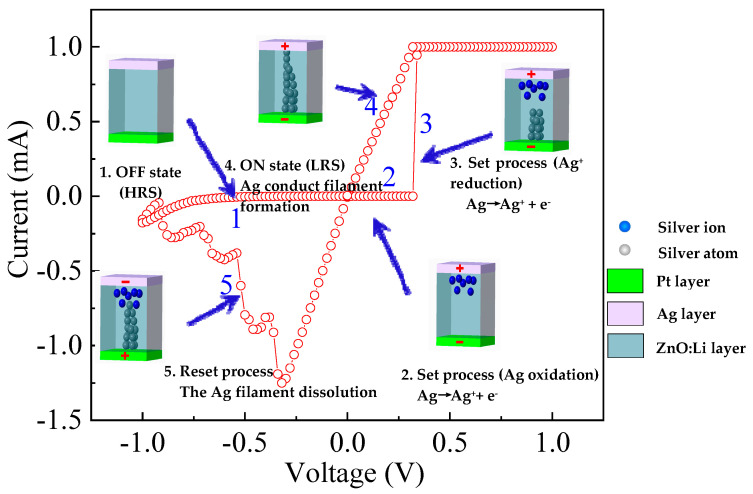
Schematic related to the formation and rupture of the metallic Ag filament in LZO-5 devices.

To further investigate the resistive switching mechanism of the LZO-5 device, an Ag conducting filament model was proposed [10,31] to analyze the formation and rupture of the metallic Ag filament in the LZO-5 device, as shown in Figure 11. Initially, when no bias voltage acted on the TE, the LZO-5 device located at HRS, as shown the step 1 in Figure 11. When exerting a positive bias, the Ag atoms are easily ionized into Ag^+^ ions under a sufficiently high electric field attributable to the higher activity of the Ag atoms [32]. After that, those resulting Ag+ ions migrated and accumulated toward the Pt cathode, as shown the step 2 in Figure 11. Finally, a conducting filament was formed between the BE and the TE. In addition, the Ag^+^ ions drifted to BE were reduced into Ag atoms at the Pt BE, confirming to step 3 in Figure 11. Therefore, the devices exhibited an ohmic transport mechanism at the LRS, as shown in step 4 in Figure 11. When applying a negative bias to the top Ag electrode, the Ag filaments were oxidized into Ag^+^ ions and then reduced at the Ag electrode, leading to the rupture of the filament conduction path corresponding to the switching of these devices from a LRS to a HRS [33], as depicted the step 5 in Figure 11.

## 4. Conclusions

In summary, a high performances Pt/Ag/LZO/Pt resistive random access memory device was designed and fabricated by using a magnetron sputtering method. The resistive switching behaviors of the Pt/Ag/LZO/Pt devices with different Li-doping contents were studied under DC and pulse voltages, respectively. The experimental results showed that the LZO-5 device, with respect to the pure ZnO and other LZO devices, presented stable bipolar resistive switching characteristics at a DC voltage, i.e., a low switching voltage less than 1.0 V, a high endurance more than 10^3^ cycles, a long retention (higher than 10^4^ s), and a large resistive switching window. In addition, quick switching between HRS and LRS can be achieved at a pulse voltage. To investigate the resistive switching mechanism of the device, a conduction mechanism model was built based on Ag conducting filament transmission. The study on the Pt/Ag/LZO/Pt device indicates that it is possible to fabricate a RRAM device with a high resistive switching performance.
